# Cerebrospinal-fluid cytokine and chemokine profile in patients with pneumococcal and meningococcal meningitis

**DOI:** 10.1186/1471-2334-13-326

**Published:** 2013-07-17

**Authors:** Leonam G Coutinho, Denis Grandgirard, Stephen L Leib, Lucymara F Agnez-Lima

**Affiliations:** 1 Departamento de Biologia Celular e Genética, Centro de Biociências, Universidade Federal do Rio Grande do Norte, Campus Universitário, Lagoa Nova, Natal, RN 59078-970, Brazil; 2 Institute for Infectious Diseases, University of Bern, Friedbuehlstrasse 51, Bern CH-3010, Switzerland

**Keywords:** Pneumococcal meningitis, Meningococcal meningitis, Cytokines, Chemokines, Interferon gamma, Cerebrospinal fluid

## Abstract

**Background:**

Bacterial meningitis is characterized by an intense inflammatory reaction contributing to neuronal damage. The aim of this study was to obtain a comparative analysis of cytokines and chemokines in patients with pneumococcal (PM) and meningococcal meningitis (MM) considering that a clear difference between the immune response induced by these pathogens remains unclear.

**Methods:**

The cyto/chemokines, IL-1β, IL-2, IL-6, TNF-α, IFN-γ, IL-10, IL-1Ra, CXCL8/IL-8, CCL2/MCP-1, CLL3/MIP-1α, CCL4/MIP-1γ and G-CSF, were measured in cerebrospinal fluid (CSF) samples from patients with PM and MM. Additionally, a literature review about the expression of cytokines in CSF samples of patients with MB was made.

**Results:**

Concerning cytokines levels, only IFN-γ was significantly higher in patients with *Streptococcus pneumoniae* compared to those with *Neisseria meningitidis*, regardless of the time when the lumbar puncture (LP) was made. Furthermore, when samples were compared considering the timing of the LP, higher levels of TNF-α (P <0.05) were observed in MM patients whose LP was made within 48 h from the initial symptoms of disease. We also observed that the index of release of cyto/chemokines per cell was significantly higher in PM. From the literature review, it was observed that TNF-α, IL-1β and IL-6 are the best studied cytokines, while reports describing the concentration of the cytokine IL-2, IL-1Ra, G-CSF and CCL4/MIP-1β in CSF samples of patients with bacterial meningitis were not found.

**Conclusion:**

The data obtained in this study and the previously published data show a similar profile of cytokine expression during PM and MM. Nevertheless, the high levels of IFN-γ and the ability to release high levels of cytokines with a low number of cells are important factors to be considered in the pathogenesis of PM and thereby should be further investigated. Moreover, differences in the early response induced by the pathogens were observed. However, the differences observed are not sufficient to trigger changes in the current therapy of corticosteroids adopted in both the PM and MM.

## Background

Bacterial meningitis (BM) is an infectious disease characterized by high mortality and morbidity rates [1]. After *Haemophilus influenza* type b immunization, *Streptococcus pneumoniae* and *Neisseria meningitidis* have become the two main causes of BM in Brazil [2-4].

The inflammatory response plays a primordial role in the pathogenesis of cerebral injury associated to different meningitis etiologies [5,6]. During BM, bacterial cell-wall components, such as lipopolysaccharide from gram-negative bacteria and lipoteichoic acid from gram-positive bacteria, trigger the massive release of proinflammatory molecules. These in turn increase the permeability of the blood–brain barrier (BBB) and attract leukocytes to the central nervous system (CNS) (pleocytosis). Cytokines, reactive oxygen species, reactive nitrogen species, and matrix metalloproteinases, act in a coordinated manner to promote an oxidative burst leading to energy failure and cell death [7-10]. The pattern of inflammatory mediators present in the cerebrospinal fluid (CSF) in response to an immune assault determines the disease’s severity and its sequelae [7,11].

BM has often been comprised in a unique group, considering that gram-positive and gram-negative bacteria compounds trigger the same Toll-like receptors (TLR). However, *S. pneumoniae* and *N. meningitidis* also seem to modulate different Toll-like receptors that consequently regulate a different expression of cytokines. TLR2, TLR4, and TLR9 are important in the response against *S. pneumoniae*, while TLR2 does not seem to be essential in the host response to *N. meningitidis*[12-15]. Conversely, important mediators of chemoattraction, such as CXCL8/IL-8, CCL3/MIP-1α and CCL2/MCP-1, induce a similar intensity of response in relation to *S. pneumonia* and *N. meningitidis*[16,17].

Although isolated studies have demonstrated higher cytokine expression during BM caused by different agents, they are limited in establishing differences between the inflammatory responses induced by each pathogen. Relevant questions, such as “are there differences between the inflammatory responses caused by different etiologic agents of BM?” remain to be answered. This comparative study is important because BM continues to have a high occurrence and the outcome from pneumococcal meningitis (PM) differs from that of meningococcal meningitis (MM) [18,19].

Currently, the BM treatment is similar for both etiological agents, i.e. antibiotics and corticosteroids. Dexamethasone remains as the antiinflammatory therapy most used during BM treatment. Clinical trials have shown reduction in unfavorable outcome, such as lower mortality in adults and fewer neurological and auditory sequelae in adults and children during PM, however similar beneficial effect was not seen for dexamethasone during MM [20]. Thus, if there is a marked difference in inflammatory profile, this may have implication for immunomodulatory therapies, which should be tailored differently for each etiology.

In this work, we measured twelve cyto/chemokines that are commonly expressed during BM in the CSF from patients with PM and MM and did a literature review about reports of cyto/chemokine determination in CSF in order to obtain further knowledge about the inflammatory profile in BM caused by meningococci and pneumococci. Since the inflammatory response has been associated to the sequelae occurrence after the disease, a better understanding of the pathophysiologic mechanisms between the host and each causative agent may contribute to designing new therapies for the reduction of death and sequelae caused by this severe disease.

## Methods

### Case selection and sample collection

Twenty-eight patients who were admitted to the Hospital Giselda Trigueiro (Natal, Brazil) with a suspicion of meningitis were enrolled in this study. This study was evaluated and approved by the Committee on Medical Ethics of the Giselda Trigueiro Hospital and by the National Committee on Ethics (CONEP) with number 0052.1.051.000-05. Informed consent was obtained from each patient participating in this study.

CSF samples were collected by lumbar puncture (LP) during standard routine for diagnosis of meningitis in the Hospital Giselda Trigueiro. The routine diagnosis includes, evaluation of clinical symptoms, detection of the pathogen in the CSF by Gram staining, positive bacterial culture, number of erythrocytes, white blood cell count (WBC) with detection of levels of polymorphonuclear granulocytes (PMN) and protein and glucose contents. Patients who had been treated with antibiotics before LP or with other diseases (such as AIDS) that affect the immune and inflammatory responses (e.g. the cytokines expression) were not included in this study. The CSF samples were collected from the initial LP at the time of admission to hospital. All samples were kept at 4°C after LP and centrifuged (3000 g, 5 min, 4°C). Supernatants were frozen and stored at -80°C until assayed. CSF was assigned a code for anonymization purpose and the study was carried out according to ethical regulations.

### Measurement of cyto/chemokine concentration

CSF cyto/chemokines levels were measured by a Bio-Plex 200 suspension array system (Bio-Rad, Hercules, CA, USA) using microsphere-based multiplex assays. In the assay, the concentration of 12 cyto/chemokines (TNF-α, IL-6, IL-1β, IFN-γ, IL-10, IL-1Ra, CCL3/MIP-1α, CCL4/MIP-1β, CCL2/MCP-1, G-CSF, CXCL8/IL-8 and IL-2) was assessed in 28 CSF samples, using the human cytokine Linco*plex* Kit (HCYTO-60 k, Lincoplex^®^, Linco Research Inc., St Charles, MA, USA). This set of cyto/chemokines was selected from literature data that report their importance during BM in patients or in animal models. Samples (25 μl) were measured undiluted and in duplicate. The assay was performed according to the manufacturer’s instructions. Samples were diluted to fit in the dynamic range of the assay when appropriate. Cyto/chemokine concentrations were calculated by Bio-Plex Manager software using a 5-parametric logistic standard curve derived from the recombinant cytokine standards provided in the kit.

For statistical analysis, samples with cyto/chemokine levels below or above the detection limits were arbitrarily assigned the values corresponding to the minimum (3.2 pg/ml) or maximum (10,000 pg/ml) limits respectively, spanning the dynamic range of the assay, and following the manufacturer’s instructions.

### Data sources

Peer-reviewed articles were searched using the Medical Literature Analysisand Retrieval System Online (National Library of Medicine, Bethesda, Maryland) and Web of Science (Thomson Reuters, New York, NY) until December of 2010, using the key words *Bacterial Meningitis* plus *cytokine* plus *patient*. Additionally, words such as interferon, interleukin, IL-1β, IL-2, IL-6, TNF-α, IFN-γ, IL-10, IL-1Ra, CXCL8/IL-8, CCL2/MCP-1, CCL3/MIP-1α, CCL4/MIP-1β and G-CSF were indentified in the retrieved articles, and reference lists of relevant studies were searched for any additional information.

### Study selection and data extraction

Original studies measuring cyto/chemokine concentrations in the CSF of living subjects diagnosed with PM and MM were included. The inclusion criteria required was the presence of the pathogen in the CSF culture or antigen agglutination tests on CSF. Studies measuring cyto/chemokine concentrations from CSF following stimulation and other diseases which can disturb the immune response were excluded to avoid bias associated with the immune challenge. The results of each relevant article were critically analyzed and data of mean, median, standard deviation (SD), standard error (SE), confidence interval (CI) and percentage of each cyto/chemokine concentration for PM and MM were extracted. Studies with qualitative results such as western blot or protein array test were not included.

### Statistical analysis

Data were analyzed using Prism 5.0 (GraphPad, San Diego, CA, USA) and tested for normality using Kolmogorov-Smirnov normality test. Almost all the variables did not fit to normal distribution and were analyzed with non-parametric methods. Differences between groups were analyzed using the one-way ANOVA non-parametric *Kruskal-Wallis test*. Data are expressed as median and (25; 75 percentiles). Pair-wise analysis between two groups was performed using the Mann Whitney test. For all the statistical tests, values of *P* < 0.05 were considered significant.

## Results

### CSF samples and cyto/chemokine measurement

In our work, *S. pneumoniae* was diagnosed in sixteen patients and *N. meningitidis* in twelve. At the time of diagnosis 5 patients with PM reported that had been sick for 12–48 hours, and 11 had been sick for >2 days. Fever, headache, neck stiffness were present in all patients during clinical diagnosis. PM was associated with brain abscess (n = 3), CSF fistula (n = 2) and acute otitis media (n = 1). Out of 16 patients with PM, 2 died and 14 were cured after standard therapy. Four patients with MM had been sick for <12 hours, 1 had been sick for 12–48 hours, and 7 had been sick for >2 days. As observed for PM, almost all patients with MM presented fever, headache, neck stiffness, mental confusion and petechias. In some cases, MM was associated with meningococcemia (n = 4). All patients with MM included in this study were cured. Overall, the majority of patients in the study were adult. Only six patients under 18 years old were included in PM and four in MM groups. CSF biochemical parameters of each causative agent, including the number of leucocytes, protein, and glucose content results, are presented in Table 1.

**Table 1:** Categorization of the meningitis etiologies according CSF routine investigation

**CSF (n samples)**	**Age**^**a**^	**Cell count**^**a**^	**Protein**^**b**^	**Glucose**^**a**^
	**(years)**	**(cells/mm**^**3**^**)**	**(g/l)**	**(mg/dl)**
*S. pneumoniae (16)*	30 (5; 53)	733 (396; 2,520)	211.6 ± 51.45	17.5 (5; 57)
*N. meningitidis (12)*	23 (7; 27)	3,360 (929; 12,925)^c^	150.4 ± 29.8	44.5 (5; 60)

^a^Values are represented as median (25; 75 percentiles); ^b^Values are represented as mean ± SE; ^c^Significant value compared to *S. pneumoniae* (*P* ≤ 0.05).

**Figure 1:**
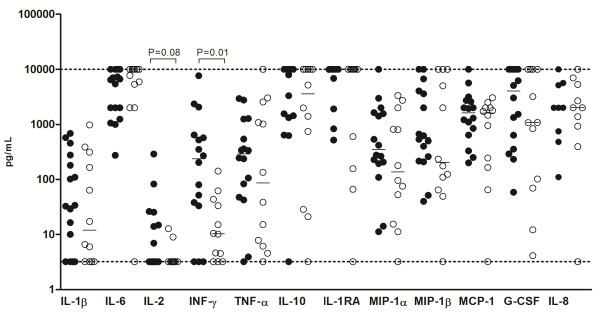
**Comparison of cyto/chemokines levels in CSF from *S. pneumoniae *and *N. meningitidis *patients. ***S. pneumoniae* patients showed similar levels of cyto/chemokines compared to *N. meningitidis* patients, except for IFN-γ (*P* < 0.05 by Mann Whitney test). IL-8 shows a lower number of samples due to CSF availability. Dotted lines mark the upper and lower limits of detection.

The comparison of the concentration of cyto/chemokines in CSF samples is shown in Figure 1. Significant differences in the levels of cyto/chemokines between PM and MM groups were not observed, except for IFN-γ. Elevated level of IFN-γ was observed in the patients with PM (median 237.2 pg/mL, range 34.5-624.7 pg/mL; *P < 0.05* ) compared to MM (median 10.33 pg/mL, range 3.5-41.1 pg/mL). Although not statistically significant, an increased level of IL-2 was also observed in patients with PM (median 3.2 pg/mL, range 3.2-22.8 pg/mL; *P = 0.08*) compared to MM (median 3.2 pg/mL, range 3.2-3.2 pg/mL). Other immune modulators, not identified or poorly identified in prior reports, such as IL-10, IL-1Ra, CCL4/MIP-1β e G-CSF, were measured and no significant difference was observed between the pathogens.

**Figure 2:**
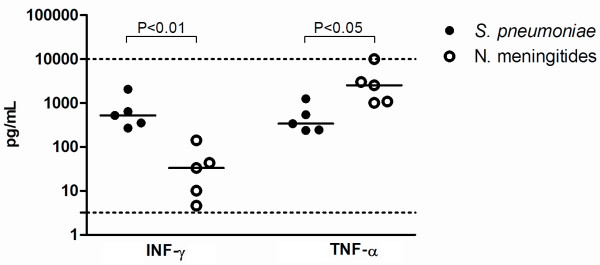
**Levels of IFN-γ and TNF-α in the CSF of patients with LP within 48 h (<48 h) from initial symptoms. ***S. pneumoniae* patients showed higher levels of IFN-γ compared to *N. meningitidis* patients (*P* < 0.01 by Mann Whitney test). The level of TNF-α in the patients with MM was significantly higher than patients with PM (*P* < 0.05 by Mann Whitney test). Dotted lines mark the upper and lower limits of detection.

**Figure 3:**
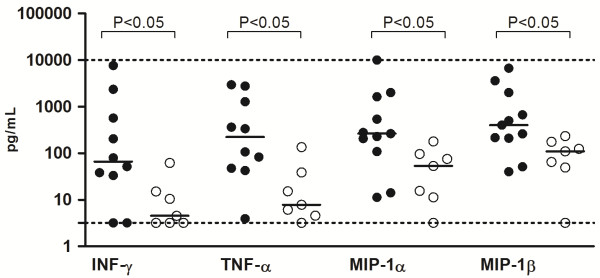
**Comparison of cytokine levels in patients with LP performed after 48 h (>48 h) from initial symptoms. ***S. pneumoniae* patients showed higher levels of IFN-γ, TNF-α, MIP-1α e MIP-1β compared to *N. meningitidis* patients (*P* < 0.05 by Mann Whitney test). Dotted lines mark the upper and lower limits of detection.

Comparative analyses of cytokines levels considering the time of symptoms prior to LP were done. Patients with LP performed within 48 hours (<48 hours) from the initial symptoms were compared to the patients with LP performed more than 48 h after (>48 h) of the onset of symptoms. Firstly, patients with PM or MM were compared separately. Among patients with PM, elevated levels (*P* <0.05) of IL-1β, MIP-1β and G-CSF were observed in group <48 h, while the patients with MM of the group <48 h showed higher levels (*P* <0.05) of IL-1β, TNF-α, IL-10, MIP-1α, MIP-1β, G-CSF, in comparison with patients of group >48 h (data not shown). Further, the comparative analyses in relation to the time of LP was done between PM and MM patients. Concerning the time <48 h, higher levels of IFN-γ were observed in patients with PM (median 521.7 pg/mL, range 311.2-1353 pg/mL; *P < 0.01* ) compared to MM (median 33.45 pg/mL, range 7.4-92.6 pg/mL), while TNF-α was significantly higher in the CSF of patients with MM (median 2549 pg/mL, range 1047–6513 pg/mL; *P < 0.05*) compared to patients with PM (median 340.4 pg/mL, range 241.5-895.5 pg/mL) (Figure 2). Between the group >48 h, PM patients showed significant higher levels of IFN-γ, TNF-α, MIP-1α and MIP-1β compared to MM patients (Figure 3). In addition, cytokine levels were compared according to the age of the patients but significant differences were not observed between children (<18 years old) and adults (>18 years old).

### Ratio between cyto/chemokine concentration and number of cell in CSF (strength of cyto/chemokine release index)

**Figure 4:**
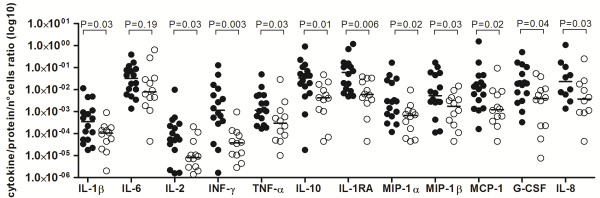
**Ratio between cyto/chemokines and the number of PMN cells during infection.** Each value of cyto/chemokine was individually divided per amount of cells. During pneumococcal meningitis (● ) cells demonstrated higher ability to release cyto/chemokines than meningococcal meningitis (○). IL-8 shows a lower number of samples due to CSF availability.

Cyto/chemokine concentrations were individually categorized by the number of cells in CSF to determine a cyto/chemokine release index. This index was indirectly used to compare the strength of activation of the immune response and the release of cyto/chemokine promoted for each pathogen. In order to avoid the interference of cyto/chemokines originated from the blood as result of the BBB permeability, CSF cyto/chemokine levels were firstly divided through the CSF protein level and then divided by the number of cells to correct the BBB disturbance. The data demonstrated a higher cyto/chemokine/cell number ratio during PM (Figure 4). As observed, PM is characterized by a significant release of chemokines, growth factors and anti inflammatory cytokines. Significant *P* values are shown in the Figure 4.

### Comparative of literature findings and cyto/chemokine concentration

A total of 282 records of cyto/chemokine studies during BM were retrieved from the literature search. Out of 282, only 7 were included in the search criteria of this study (Table 2). Notably, selected reports showed the concentration of TNF-α comprises 6/7 studies, following IL-6, IL-1β and IFN-γ with 2/7 studies, and only 1/7 of the retrieved reports measured the concentration of IL-10, IL-1Ra, CXCL8/IL-8, CCL2/MCP-1 and CCL3/MIP-1α. Reports showing the concentration of IL-2, IL-1Ra, CCL4/MIP-1β and G-CSF in CSF of human patients during PM or MM were not observed in this literature screening. TNF-α howed a contradictory result in the different studies, 2/6 of them demonstrated elevated levels of TNF-α during meningitis caused by *S. pneumoniae,* while 4/6 demonstrated the highest levels during NM [17,21-26]. In the two reports that IFN-γ was measured, the values were significantly higher in meningitis caused by *S. pneumoniae* than by *N. meningitides*[23,25]*.* Mastroianni *et al*. [17] observed similar levels of CXCL8/IL-8 and CCL3/MIP-1α in meningitis caused by both studied pathogens, although CCL2/MCP-1 was higher in patients with NM. The cytokine IL-6 levels were observed in two studies, but they were controversial and not conclusive, due to the small number of patients included in each study [21,24].

**Table 2:** Comparative literature screening of cytokine measurements in CSF from pneumococcal and meningococcal meningitis patients

**IL-1β**		**TNF-α**		**IL-6**		**IFN -γ**		**IL-10**		**MIP-1α**		**MCP-1**		**IL-8**		**References**
***S.p***	***N.m.***	***S.p.***	***N.m.***	***S.p.***	***N.m.***	***S.p.***	***N.m.***	***S.p.***	***N.m.***	***S.p.***	***N.m.***	***S.p.***	***N.m.***	***S.p.***	***N.m.***	
2988 (3)	268 (3)	4238 (3)	57 (3)			2.5 (u/ml) (3)	<0.1 (u/ml) (3)									[25]
320 (10)	300 (18)	450 (10)	500 (18)													[26]
		584 (14)	2827 (7)													[22]
		2324 (6)	414 (13)			100% (6)	46% (13)	736 (6)	654 (13)							[23]
		140 (2)	2007 (1)	8225 (4)	4500 (1)											[21]
		23.69 (2)	86 (4)	9.36 (2)	44.8 (4)											[24]
										23 (7)	25 (5)	3145 (7)	4604 (5)	4454 (7)	4525 (5)	[17]

*S.p*. *Streptococcus pneumonia*, *N.m. Neisseria meningitides,* Values without units are in pg/mL. Parenthesis shows the number of patients included in each study.

Almost all studies showed elevated cyto/chemokine concentrations in BM patients when compared with healthy subjects. The works included a variety of patients of different ages, 4/7 studies showed cytokines levels in infants, children and adolescents [21,23-25], while 2/7 included only adult patients [22,26] and 1/7 studies included both children and adults [17]. The amount of patients included in each study was distinct, in 4 of 7 studies the range was 1 to 12 individuals for both pathogens [17,23,26], while 3/7 had from 19 to 28 individual for both pathogens [17,18,21]. Only one study described the presence of neurological sequelae, including hearing loss and cerebral atrophy, relating to the pathogen [25]. Data of IL-1β, IL-6, TNF-α, IFN-γ, IL-10, CXCL8/IL-8, CCL2/MCP-1 and CCL3/MIP-1α concentrations were measured from CSF patients using only two types of techniques, ELISA (5/7) and immunoradiometric assays (2/7).

## Discussion

Different experimental approaches have been used to analyze the expression levels of the immune modulators, such as microarray and ELISA assays. In general, studies have mainly focused on the comparison of BM and meningitis caused by other infectious agents such as viruses and fungi, using healthy individuals as control [27,28]. However, the comparative analysis in relation to the levels of cytokines expressed during PM and MM has been little explored. The literature screening revealed the need of more comparative studies, in particular for cyto/chemokines poorly studied in BM patients, such as IL-10, CXCL8/IL-8, CCL3/MIP-1α and CCL2/MCP-1. To our knowledge, our work is the first to assess the levels of cytokines IL-2, IL-1Ra, CCL4/MIP-1β and G-CSF in patients with PM and MM. In contrast, some cytokines have been well characterized in BM such as IL-1β, TNF-α and IL-6, which have received more attention in the majority of the studies. In general, a limited profile of cytokine expression from the two major meningitis-pathogens was observed in each study. The use of the multiplex method in our work allowed the comparison between patients with PM and MN with a broader spectrum of cytokines analyzed simultaneously.

In all analyzed studies, including ours, some limitations are observed. Among these, the small number of patients and a lack of correlation between cytokines levels and symptoms of the disease and its sequelae after treatment, which may be related to some aspects of BM. Collection of CSF is an invasive procedure, therefore obtaining the samples is usually associated with routine diagnosis of the disease, which generally only occurs in emergency situations. In addition, poor hospital conditions in the public health system of developing countries, where BM cases occur more frequently, make it difficult access to patients’ clinical data.

TNF-α is one of the most studied cytokine during BM and has been identified as a useful complementary tool to improve the diagnosis of meningitis, especially in cases where the CSF examination is inconclusive. Although this cytokine seems to be important in driving several mechanisms underlying the immune response, our experimental data and the literature screening showed that this cytokine is not differentially expressed during meningitis caused by *S. pneumoniae* or *N. meningitidis*[22,24,27]. However, in relation to the time of LP, in the group <48 h we observed that the TNF-α levels are significantly elevated in CSF of patients with MM, but the same result is not repeated when LP was performed after 48 h from onset of symptoms. This data suggest a rapid decline of TNF-α during MM. This observation is in agreement with previous works. Van Deuren et al. [29] analyzed the kinetics of the TNF-α release in the animal models of meningococcal disease and sepsis and observed that TNF-a reached the maximal level in 1–2 h after LPS injection, becoming undetectable after 18–24 h. In human patients, the highest level of TNF-α can be detectable in the first 48 h of disease onset, decreasing after this period, which suggest that after a proinflammatory state, a down regulation of TNF-α production may represent a transition to an anti‒inflammatory state as a protective mechanism [30-32].

The IL-6 levels are also elevated during BM and some researchers have tried to associate this with TNF-α for the differential diagnosis of BM, but without success until now. Dulkerian *et al*. [21] and Mukai *et al*. [24] observed high levels of IL-6 during BM without differences between PM and MM. Other cytokines or chemokines, such as IL-1β, IL-10, CXCL8/IL-8, CCL3/MIP-1α and CCL2/MCP-1 did not demonstrate any significant difference between pathogens [17,23,26,27]. Corroborating with these authors, our measurements also did not show differences between pathogens for these cytokines.

It is plausible that additional factors can lead to differences in the cytokine expression, such as the type of the pathogen strain, the host’s age and the time of the LP after infection (as it was observed in our data). Strains of *N. meningitidis* seem to be able to induce different cytokine profiles [33,34]. Considering the age of the patients, Sharief *et al*. [26] observed that concentrations of TNF-α and IL-1β in CSF of patients with bacterial meningitis were not age-dependent. Additionally, Dulkerian *et al*. [21] demonstrates that neonates and young infants respond to pathogen invasion with the release of cytokines at comparable levels to those found in older children and adolescents. Similarly in our study, age-related differences were not observed (data not shown). However, in relation to the time of LP, we observed some differences between the pathogens. The MM patients seem to have a decay of cytokine (such as TNF-α, IL-1β, IL-10, MIP-1α, MIP-1β and G-CSF) levels faster than patients with PM, since decreased levels of certain cytokines were observed in the group >48 h.

IFN-γ was the unique protein that showed higher concentration in patients with PM compared with MM independent of the time of the LP. Kornelisse *et al*. [23] observed that IFN-γ was significantly higher in patients with PM than in children with meningitis caused by *H. influenza* or *N. meningitidis* and obtained data suggesting that the production of IFN-γ in the CSF from patients with bacterial meningitis is induced by IL-12, with TNF-α as a co-stimulator. The high IFN-γ production in monocytes culture seems to be mainly attributed to Gram-positive bacteria, which induce much more IL-12 than Gram-negative bacteria [35].

IFN-γ is involved in the stimulation of non-specific defense mechanisms of macrophages and polymorphonuclear leukocytes, such as phagocytosis and secretion of reactive oxygen intermediates [36]. Furthermore, our data also showed that IL-2 seems to be more pronounced in PM than in MM. The highest expression of IFN-γ and IL-2 during PM suggests the occurrence of a Th1 immune response [37]. In fact, TH1 and TH17 response were observed in a co-culture model of human monocytes and CD4^+^T cells stimulated with *S. pneumoniae*[38].

The difference in the degree of immune response observed during pneumococcal infection may be related to the best or worst outcome of the disease [39]. A deeper understanding of the immune response pathways against each pathogen, such as the type of Th response, can help to develop adjunctive immunotherapy using Th-polarized cytokines to increase host defense and accelerate healing of the pathogen.

Patients with PM, even with significantly lower numbers of WBC as demonstrated in Table 1, possess cyto/chemokines concentration at the same levels of patients with MM. Normalized concentrations of inflammatory mediators by cell count suggest a higher degree of activation on a cellular level, mainly during meningitis caused by *S. pneumoniae* (Figure 4). The difference in the IFN-γ concentration can be reflected in the potential of cells to release inflammatory cytokines, or this mediator can work as a signal to regulate invading leukocytes during BM [40].

The high immunogenicity of *S. pneumoniae* caused by cell components, e.g., pneumolysin, lipoteichoic acid and nucleic acids, can be responsible to release an elevated amount of cytokines per cell number [41,42]. Moreover, anti-inflammatory cytokines such as, IL-1Ra and IL-10 demonstrated the highest values to this index of cell activation. These inflammatory modulators could be blocking the recruitment of other cells. Although IL-8 and MCP-1 are important to attract phagocytic cells and have been detected at similar levels in both pathogens, the number of cells was significantly higher during MM than PM. Some studies have shown that IL-8 from human and rabbits is not able to induce pleocytosis [43,44]. In addition, pleocytosis has not been shown to be directly correlated to a specific chemokine, but can be correlated to the shift of the type of invading leukocyte [17,40,45]. No correlation between the chemokines IL-8, MCP-1 and MIP-1α levels and the total number of leucocytes in the CSF during BM was found. Even when separately comparing either CSF concentrations of IL-8 with neutrophil counts, or MCP-1 and MIP-1α with mononuclear cells in the CSF, no correlation was observed [17].

Even though the amount of cytokines and chemokines by cell counts differ between pathogens, the overall profile of these mediators in the CNS seems to be very similar. However, based on the IFN-γ, TNF-α and IL-2 found in this study, *S. pneumoniae* can trigger a Th1 response, differing from *N. meningitidis* in this aspect, which needs to be better investigated. This information about T helper response may also facilitate the design of meningitis vaccines based on cell-mediated immunity and provide new approaches based on the susceptibility of each patient to the disease.

BM outcome is correlated with severity of the inflammatory response in the subarachnoid space, thus, it is clear the importance of steroid therapy during BM. However, the benefit of adjunctive dexamethasone for all or any subgroup of patients with bacterial meningitis remains unproven [20,46]. Considering the data on cytokine levels, no changes in the regimen of dexamethasone for PM or MM would be need. However, different pathogens seem trigger a heterogeinity of type and degree of immune response, thus, advances in the pathogen-specific anti-inflammatory therapy are needed in order to better prognosis for BM patients.

## Conclusions

In this work, the basic knowledge of the expression of several cyto/chemokines during PM and MM was increased. The modulation of cytokine levels in meningitis of diverse bacterial etiologies may be a useful strategy to improve the outcome in terms of morbidity and mortality. However, other inflammatory proteins that could play important roles in the steps of immune response were not included in the present study. In the future, more systematic studies containing a greater number of individuals, expanding ranges of cytokines and associating with time, sequels and the outcome of meningitis disease should be done to contribute to a more detailed understanding of the pathogenesis underlying meningitis.

## Abbreviations

BM: Bacterial meningitis; CSF: Cerebrospinal fluid; PM: Pneumococcal meningitis; MM: Meningococcal meningitis; WBC: White blood cell count; LP: Lumbar puncture; CNS: Central nervous system; PMN: Polymorphonuclear granulocytes; IL: Interleukin; CXCL: Chemokine (c-x-c motif) Ligand; MCP: Monocyte Chemotactic Protein; CCL: Chemokine (C-C motif) Ligand; MIP: Macrophage Inflammatory Protein; TNF-α: Tumour Necrosis Factor-alpha; IFN-γ: Interferon-gamma; G-CSF: Granulocyte Colony-Stimulating Factor.

## Competing interests

The authors declare that they have no competing interests.

## Authors’ contributions

LC performed the experiments, analyzed the data, and drafted the manuscript. DG aided in performing the experiments. SL designed the study and revised the manuscript. LA designed the study, analyzed the data, and revised the manuscript. All authors read and approved the final manuscript.

## Pre-publication history

The pre-publication history for this paper can be accessed here:

http://www.biomedcentral.com/1471-2334/13/326/prepub
